# Affective-cognitive circuits in postoperative appetite reduction: an adaptive neuroimmune response to surgical stress

**DOI:** 10.3389/fnins.2025.1654559

**Published:** 2025-08-12

**Authors:** Yanbo Sun, Xianrong Bao, Yunyun Cen, Huiyin Wu, Feng Sun, Lin Fu

**Affiliations:** ^1^Center for Life Sciences, Yunnan Key Laboratory of Cell Metabolism and Diseases, School of Life Sciences, Yunnan University, Kunming, China; ^2^Department of Gastrointestinal Surgery, The Second Affiliated Hospital of Kunming Medical University, Kunming, China; ^3^General Surgery Department, Weixin County People's Hospital, Zhaotong, China; ^4^Key Laboratory of Tumor Immunological Prevention and Treatment in Yunnan Province, Yan'an Hospital Affiliated to Kunming Medical University, Kunming, China

**Keywords:** feeding behavior, appetite perception, inflammatory responses, postoperative, neuroimmune factors

## Abstract

Postoperative reduction in appetite perception, conceptualized as an interplay between emotion, perception, and cognition, may lead to adverse nutritional outcomes. However, an increasing body of research suggests that it may serve as an adaptive mechanism to inhibit inflammatory responses and regulate metabolic burden. This review comprehensively summarizes the multifaceted mechanisms underlying postoperative changes in appetite perception, particularly from the perspectives of immune regulation, inflammatory suppression, and metabolic reprogramming. Special attention is paid to the affective and cognitive dimensions of appetite perception, exploring how emotion-related processing and neurocognitive feedback contribute to appetite perception suppression during recovery. Moreover, this review highlights the clinical significance of these affective-perceptual changes in postoperative nutritional management, emphasizing the need to integrate psychological, perceptual, and neuroimmune factors into patient care strategies. Ultimately, the article explores the potential role of postoperative appetite perception reduction in modulating insulin sensitivity and improving systemic metabolic health. Based on current literature, we advocate for reevaluating appetite perception dynamics during recovery to provide novel theoretical foundations and practical directions for targeted postoperative nutritional interventions.

## 1 Introduction

Postoperative reduction in appetite perception is a prevalent clinical phenomenon, with most patients undergoing major abdominal surgery experiencing a significant loss of appetite perception between 48 h after surgery and discharge. The incidence ranges from 30 to 60%, with rates exceeding 70% among the elderly, females, and patients undergoing gastrointestinal or orthopedic surgery (Wagner et al., 2022; Nguyen et al., 2023). Traditionally viewed as a passive adaptive response of the body to surgical trauma and inflammatory reactions (Wagner et al., 2022). However, emerging evidence suggests that this phenomenon involves more than a physiological adjustment; it may also represent an affective-cognitive adaptation driven by both peripheral inflammation and central perception mechanisms (Shields et al., 2017). Specifically, appetite perception combines emotional, motivational, and cognitive factors, acting as a key regulator in the postoperative stress response.

Recent studies have increasingly recognized that the reduction in appetite perception may serve as a protective, self-regulatory mechanism. The core mechanisms involve metabolic reprogramming, neuroendocrine regulation, and immune network remodeling, with perception and emotion playing integral roles in modulating feeding behavior and inflammatory responses. This adaptive mechanism may assist patients in recovery by reducing metabolic demands and alleviating inflammatory burdens.

Modern medicine often associates surgery with complex pathophysiological stress responses, especially those involving inflammation and immune activation. Surgical trauma induces the release of pro-inflammatory mediators, such as C-reactive protein (CRP), Tumor necrosis factor alpha (TNF-*α*), Interleukin-6 (IL-6), and Interleukin-1β (IL-1β), which alter immune cell metabolism and affect the cognitive-affective regulation of appetite *via* neuroimmune circuits.

When peripheral inflammation occurs, pro-inflammatory factors send inflammatory signals to the brain through two quick pathways. First, they project to the nucleus of the solitary tract in the brainstem via the vagus nerve and sensory fibers in the spinal cord (Huerta et al., 2025; Murphy et al., 2023), followed by the upward regulation of the arcuate nucleus (ARC) (de Morentin et al., 2024) and paraventricular nucleus (PVN) (Murphy et al., 2023). Second, they bypass barrier-like areas such as the choroid plexus or median carina of the blood–brain barrier, or they directly alter the permeability of the blood–brain barrier (Gryka-Marton et al., 2025). This allows cytokines and their receptors to enter the hypothalamic tissue. Once inside the central nervous system, these signals activate the nuclear factor kappa-light-chain-enhancer of activated B cells (NF-κB) and the janus kinase/signal transducer and activator of transcription 3 / protein kinase B (JAK-STAT3) pathways in microglia and astrocytes (Lawrence et al., 2023; Lee and Dixit, 2017), leading to the release of secondary inflammatory mediators like IL-1β (Mirtella et al., 1995). This inhibits neuropeptide Y / agouti-related peptide (NPY/AgRP) neurons (Chaskiel et al., 2019) and increases the excitability of proopiomelanocortin (POMC) and cocaine and amphetamine-regulated transcript (CART) neurons (Grossberg et al., 2010), quickly decreasing appetite perception. At the same time, pro-inflammatory factors boost leptin secretion from fat cells (Faggioni et al., 1998). Elevated leptin then acts on the leptin receptor in the ARC (Tan et al., 2024), which suppresses eating in the short term. However, ongoing inflammation weakens leptin signaling through mechanisms like the suppressor of cytokine signaling (SOCS3) overexpression, turning a state of high leptin and low appetite into leptin resistance (Wunderlich et al., 2013), worsening appetite loss.

In turn, appetite reduction decreases the synthesis of pro-inflammatory factors by inhibiting mitochondrial function and glycolytic pathways in immune cells. Meanwhile, reduced postoperative energy intake promotes macrophage polarization toward the M2 anti-inflammatory phenotype by downregulating mechanistic target of rapamycin complex 1 (mTORC1), enhancing Interleukin-10 (IL-10) secretion, and modulating emotional and cognitive signals linked to appetite suppression.

Appetite perception thus emerges as not merely a behavioral response, but a reflection of integrated cognitive and emotional processing under stress. This review further explores the neuroendocrine-immune synergy that contributes to central appetite inhibition through hypothalamic–pituitary–adrenal (HPA) axis activation, elevated cortisol levels, and suppression of hypothalamic NPY/AgRP neurons. These neurohormonal processes suppress pro-inflammatory factor secretion *via* glucocorticoid receptor (GR)-mediated NF-κB inhibition, reinforcing the cognition-perception-immune feedback loop (Tucker et al., 2024; Zhao et al., 2024).

Adipose tissue, as a metabolic, immune, and affective interface, downregulates leptin and STAT3 signaling while increasing IL-10 and promoting anti-inflammatory macrophage activity (Barrios et al., 2022; Kiernan et al., 2023; Wang et al., 2023). Gut-derived signals such as ghrelin, Glucagon-like peptide-1 (GLP-1), and peptide YY (PYY) also participate in appetite regulation through intertwined cognitive-emotional and inflammatory pathways: ghrelin is suppressed to promote autophagy *via* adenosine monophosphate-activated protein kinase (AMPK)/ mTOR, GLP-1 delays gastric emptying and inhibits NF-κB, and PYY enhances intestinal integrity *via* Y1-mediated immune signaling (Chen et al., 2020; Pierre et al., 2023).

Skeletal muscle metabolic reprogramming and mitochondrial dynamics (*via* AMPK/peroxisome proliferator-activated receptor *γ* coactivator l alpha (PGC-1α) and reactive oxygen species (ROS)-NF-κB axes) also impact systemic inflammatory responses and appetite-related affective states (Irazoki et al., 2023; Valentine et al., 2020). The gut microbiota gut-brain axis further integrates peripheral immune signals with central emotional and cognitive processing, suggesting its critical role in postoperative appetite regulation. Future research should continue to explore selective GR modulators and targeted neuroimmune interventions, providing mechanistic insight for personalized rehabilitation (Butts and Sternberg, 2008; van de Wouw et al., 2017).

**Figure 1:**
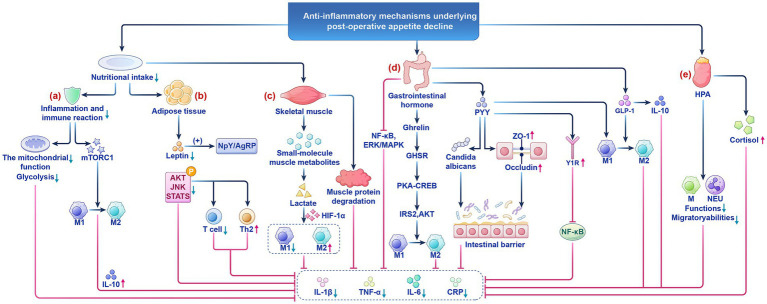
The anti-inflammatory mechanism of postoperative appetite reduction. **(a)** Low nutritional status suppresses the mitochondrial function of immune cells and glycolytic pathways. **(b)** Adipose tissue is both a core organ for energy storage and a key hub for regulating systemic metabolism and immune balance. **(c)** The pathological coupling of postoperative low metabolism and skeletal muscle consumption is key to patient recovery. **(d)** Gastrointestinal hormones such as Ghrelin, GLP-1, and PYY in appetite perception regulation and inflammatory responses. **(e)** The HPA axis plays a central role in postoperative metabolic and immune homeostasis through dual regulatory mechanisms. HPA, the hypothalamic pituitary adrenal; M, Macrophages; MEU, Neutrophils.

Based on this, this study systematically reviews high-quality literature to explore the affective-cognitive mechanisms and perception-centered immune-metabolic crosstalk underlying the reduction of postoperative appetite perception (Figure 1). Through the synergistic integration of perception, emotion, metabolism, and immunity, we aim to promote a transition from empirical to precision medicine and establish a new paradigm for optimizing patient prognosis. This review will first dissect the inflammatory underpinnings, then explore the neuroendocrine-immune crosstalk, and finally discuss clinical translation.

## 2 Inflammatory mechanisms of postoperative reduction in appetite perception

### 2.1 Postoperative reduction in appetite perception and inflammation

The regulatory mechanisms of postoperative reduction in appetite perception and inflammation reveal the complex adaptive responses of the body under trauma stress. This process involves metabolic reprogramming, neuroendocrine regulation, and immune network remodeling, forming a multidimensional regulatory system of inflammation suppression-tissue repair-metabolic balance, providing an endogenous protective mechanism for postoperative recovery (Moschen et al., 2010).

**Figure 2:**
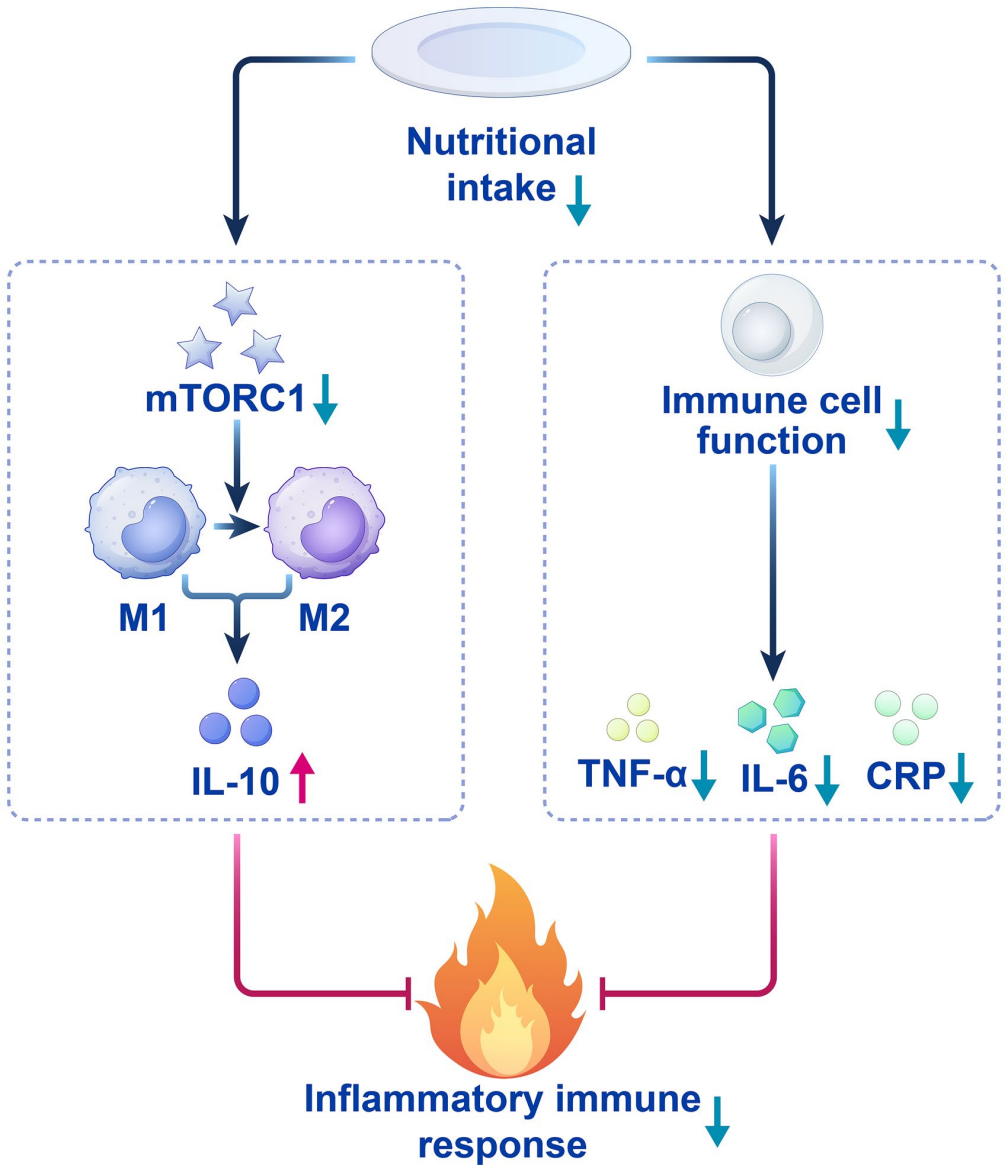
The anti-inflammatory mechanism of low nutrient intake.

CRP, as a core biomarker of postoperative inflammation, has a negative correlation with low nutritional intake. Low nutritional status suppresses the mitochondrial function of immune cells and glycolytic pathways, thereby reducing CRP secretion and alleviating systemic inflammatory responses (Khan et al., 2023; Martindale, 2023) (Figure 1a). Moreover, the synthesis rate of pro-inflammatory cytokines also decreases (Hu et al., 2024; Procaccini et al., 2024). This process both helps control systemic inflammatory responses and provides a favorable environment for local tissue repair. Additionally, reduced nutritional intake promotes macrophage polarization toward M2 by downregulating the mTORC1 signaling pathway, thereby increasing the secretion of the anti-inflammatory factor IL-10 (Linke et al., 2017). IL-10 may be upregulated during postoperative reduction in appetite perception, further inhibiting pro-inflammatory responses and reducing potential side effects caused by surgical trauma (Nagata and Nishiyama, 2021; Stein et al., 2018; Tiwari et al., 2024). The dynamic balance changes between pro-inflammatory factors and anti-inflammatory factors caused by low nutritional intake help explain the potential protective effect of decreased appetite perception after surgery (Figure 2).

Postoperative reduction in appetite perception may provide an endogenous, proactive inflammatory regulatory mechanism by directly or indirectly modulating inflammatory factor levels. This mechanism plays a positive role in inhibiting excessive inflammatory responses, promoting tissue repair, and accelerating overall recovery. Therefore, exploring the relationship between appetite perception reduction and inflammatory factors helps elucidate the potential mechanisms of postoperative recovery, and provides new perspectives for clinical interventions.

### 2.2 Adipose metabolism and the inhibition of pro-inflammatory factors

Adipose tissue is both a core organ for energy storage and a key hub for regulating systemic metabolism and immune balance (Figure 1b). It participates deeply in the dynamic balance of postoperative inflammatory responses by secreting adipokines (such as leptin, adiponectin) and regulating the functions of immune cells (such as macrophages, T cells) (Sakers et al., 2022). Early postoperative inflammatory signals triggered by surgical trauma activate the immune cell network within adipose tissue, leading to a cascade release of inflammatory mediators. This process may induce systemic inflammatory responses while clearing infections, increasing the risk of postoperative complications (Chen et al., 2021; Cooper et al., 2021).

Immune cells such as macrophages, T cells, and B cells in adipose tissue play important roles in early postoperative inflammatory responses (Villarreal-Calderon et al., 2021). For instance, macrophages exhibit high plasticity and can polarize into M1 (pro-inflammatory) or M2 (anti-inflammatory) types. The postoperative inflammatory microenvironment activates M1 macrophages through the Toll-like receptor 4 (TLR4)/NF-κB pathway, causing them to secrete pro-inflammatory factors while inhibiting the expression of anti-inflammatory factors such as adiponectin (Boronat-Toscano et al., 2022). These factors play dual roles in the postoperative recovery process: on one hand, they help clear infections and repair damage; on the other hand, excessive activation may trigger systemic inflammatory responses, interfering with metabolic stability and exacerbating the occurrence of postoperative complications (Wacker et al., 2023).

Postoperative reduction in appetite perception is an important response of the body to trauma, often accompanied by reduced energy intake. It regulates adipocyte metabolism and inflammatory status *via* a multi-pathway regulatory network, inhibiting leptin secretion (Table 1). Energy restriction reduces lipolysis in adipocytes, leading to a 40–60% decrease in leptin levels, which activates arcuate nucleus neurons in the hypothalamus to inhibit NPY / AGRP neuron activity, forming a negative feedback regulatory loop of leptin secretion (Tucker et al., 2024; Zhao et al., 2024). Leptin is not only a marker of energy status, but also a regulatory factor of inflammatory responses in adipose tissue (Monteiro et al., 2019). For example, the reduction of leptin in adipocytes can inhibit phosphorylation of the c-Jun N-terminal kinase (JNK)/STAT3/protein kinase B (AKT) signaling pathway, attenuating the polarization of M1 macrophages, reducing T cell proliferation, promoting the immune response towards the TH2 phenotype, and inhibits STAT3 phosphorylation, reducing the activation of pro-inflammatory signals, thereby lowering the expression of pro-inflammatory factors (Barrios et al., 2022; Kiernan et al., 2023; Wang et al., 2023). Therefore, postoperative reduction in appetite perception is both an adaptive adjustment of energy reserves and an active protective mechanism achieved through the adipose-immune-neuro axis.

**Table 1:** Immune regulation of adipose and muscle metabolism.

Tissue	Model / Objective	Clinical outcomes	References
Adipose	Diet-induced obese mice	Calorie restriction → Adipocyte lipolysis ↓ → Leptin ↓ → Weight loss	Zhao et al. (2024)
	The Central or peripheral nervous system	Leptin-ARC negative feedback loop → LepR neuron → inhibits NPY / AgRP	Tucker et al. (2024)
	Multiple immune cells (Macrophages, T cells, etc.)	Leptin is a bridge between fat and the immune system	Monteiro et al. (2019)
	Neutrophilic airway inflammation mouse model	Leptin ↓/ LepR antagonism → JNK / STAT3 / AKT phosphorylation ↓ → Inhibition of M1 polarization	Wang et al. (2023)
	CD4-Cre LepR^fl / fl^ mice with a high-fat diet (HFD) for 18 weeks	T cell-specific LepR deletion → Th1 / Th17 ↓, Treg ↑	Kiernan et al. (2023)
Muscle	Muscle-specific NF-κB suppression (MISR)	AMPK / PGC-1α → Inhibition of mTORC1 → paracrine IL-6 in skeletal muscle ↓	Valentine et al. (2020)
	Different immune cells	Postoperative low metabolism + Calorie restriction (CR) → Muscle metabolites ↓ → Immune activity	Hu et al. (2024) and Oswald et al. (2024)
	(Tumor-associated) macrophage	Lactate → HIF-1 α axis: Inhibits M1, drives M2 polarization & angiogenesis	Ivashkiv (2020) and Xu et al. (2024)
	Bone metabolism	β-aminoisobutyric acid (BAIBA) → AMPK activation → NF-κB ↓ / IL-1β ↓	Yi et al. (2023)
	Elderly patients undergoing bariatric surgery	Low activity + inflammation synergy → Anabolic resistance, abnormal accumulation of muscle glycogen / lipids	Stumpf et al. (2025)

## 3 Postoperative reductions in appetite perception and immune regulation of muscle metabolism

Postoperative reduction in appetite perception is often accompanied by the formation of a low metabolic state in the body (Arai et al., 2023; Picca et al., 2022). The pathological coupling of postoperative low metabolism and skeletal muscle consumption is key to patient recovery (Figure 1c) (Table 1). For instance, skeletal muscle metabolic reprogramming through the AMPK/PGC-1α pathway inhibits mTORC1 activity, reduces IL-6 paracrine secretion, and regulates mitochondrial dynamics to influence ROS generation and NF-κB nuclear translocation (Irazoki et al., 2023; Valentine et al., 2020). Studies have found that postoperative patients often experience reduced appetite perception and insufficient energy intake, closely related to systemic inflammatory responses. The low metabolic state caused by postoperative reduction in appetite perception may reduce the release of muscle metabolic products and regulate the activity of immune cells (Hu et al., 2024; Oswald et al., 2024), thereby alleviating local and systemic inflammatory responses. *β*-aminoisobutyric acid (BAIBA) is a product of valine and thymine catabolism. It can downregulate NF-κB and reduce the production of IL-1β by macrophages (Yi et al., 2023).

Another similar skeletal muscle metabolite is Lactate, as a core molecule released by muscle metabolism, can mediate hypoxia-inducible factor 1 alpha (HIF-1α) to inhibit the activation of M1 pro-inflammatory macrophages and promote the polarization of M2 macrophages towards an anti-inflammatory and pro-angiogenic phenotype (Ivashkiv, 2020; Xu et al., 2024). And some studies have shown that lactate can pass through monocarboxylate transporters (MCTs), particularly MCT1 and MCT2, in the brain capillary endothelium to cross the blood–brain barrier and enter metabolic sensing areas such as the hypothalamus (Pierre and Pellerin, 2005). Once inside the brain, lactate can be taken up by glucose-sensitive neurons in the arcuate nucleus and converted into energy signals to regulate neuronal excitability (Cortes-Campos et al., 2013; Órdenes et al., 2021). Furthermore, lactate can regulate energy metabolism and the inflammatory state by activating its receptor, G-protein-coupled receptor (GPR81), which is expressed in the hypothalamus and periventricular zone, thereby participating in central appetite regulation (Liu et al., 2009). Therefore, lactate is not only an energy substrate, but also a metabolic signalling molecule which may inhibit appetite *via* the lactate-MCT-hypothalamus pathway under postoperative or stressful conditions. This constitutes an important link in the muscle-brain metabolic dialogue.

Recent nutritional-metabolic studies have shown that, under non-nutritional deficiency conditions, short-term appetite perception reduces or mild to moderate caloric restriction can directly weaken the inflammatory signals in skeletal muscle cells. And evidence from both humans and rodents suggests that when the body is in an energy-deficient state, myogenic IL-6 acts as an energy distribution factor, temporarily down-regulating immune activity within the muscle to save ATP for maintaining basic contractile function (Kistner et al., 2022). Additionally, in rats, continuous 30–40% energy restriction significantly inhibits the activity of the muscle NF-κB-TNF-*α* axis and downregulates the transcription of inflammatory genes such as *Tnf-α* and *Il-6* (Hernández-Saavedra et al., 2021). Moreover, 24-h fasting or alternate-day fasting in rats can reduce the levels of IL-1β, IL-6, and TNF-α in skeletal muscle and the periphery, suggesting that energy deficiency itself can trigger an “anti-inflammatory program” (Speaker et al., 2016). Numerous studies have shown that energy restriction activates the silent information regulator of transcription (SIRT)-AMPK pathway and inhibits the NF-κB/NOD-like receptor protein 3 (NLRP3) inflammasome, making it a potential intervention strategy for various inflammatory diseases (Kökten et al., 2021). In the CALERIE study and its subsequent follow-up, after 2 years of 25% caloric restriction in human subjects, the overall expression of inflammatory-stress genes in skeletal muscle was down-regulated. Still, muscle strength did not significantly decrease (Das et al., 2023).

Therefore, the early postoperative appetite perception was suppressed, and relatively low nutritional intake may temporarily reduce the local inflammatory load in the muscle through pathways such as NF-κB inhibition, SIRT-AMPK activation, and IL-6 energy-immune redistribution, providing time for tissue repair and energy reconstruction. However, as wound healing progresses and metabolic demands increase, protein-energy supply should be restored promptly to avoid long-term negative balance leading to muscle atrophy and functional decline.

## 4 Postoperative reduction in appetite perception and immune system regulation

The low metabolic state results in a postoperative reduction in appetite perception that regulates immune system function through multidimensional mechanisms, forming a complex adaptive protective mechanism. In this state, the body reprograms energy metabolism and regulates neuro-immune interactions to inhibit the activity of immune cells, thereby alleviating postoperative inflammatory responses. For instance, the functions, migratory abilities, and interactions of key immune cells such as macrophages and neutrophils are significantly reduced, leading to decreased release of pro-inflammatory factors. Simultaneously, the sensitivity of immune cells to inflammatory signals is diminished, reducing excessive responses to surgical trauma (Shafqat et al., 2023; Viola et al., 2019). This immune suppression effect may be an adaptive response of the body, preventing excessive activation of the immune system that could lead to complications, thus creating a favorable environment for postoperative recovery.

Research indicates that the postoperative low metabolic state may inhibit immunity through two pathways: on one hand, surgical trauma and stress responses lead to elevated glucagon, cortisol, and pro-inflammatory hormones, promoting glycogenolysis, and the increased cortisol leads to nutrient loss and weakening immune cell function (Hirsch et al., 2021; Ivascu et al., 2024). On the other hand, pro-inflammatory factor IL-6 exhibits a bidirectional regulatory effect under metabolic constraints, driving initial inflammatory responses while inducing immune-suppressive cells. As a pro-inflammatory cytokine present in nutritional stress and chronic inflammatory states, IL-6 paradoxically promotes the accumulation and immunosuppressive activity of myeloid-derived suppressor cells (MDSCs), maintaining immune balance (Bobbo et al., 2021; Šestan et al., 2024; Sharma et al., 2021; Zeng et al., 2024).

In conclusion, postoperative reduction in appetite perception is not merely a passive response to surgical trauma but may represent a complex adaptive protective mechanism. The body can effectively control postoperative inflammatory responses by inducing a low metabolic state, suppressing immune cell activity, and reducing the sensitivity of pro-inflammatory signals. These studies provide new directions for postoperative interventions, particularly in regulating immune responses through nutritional management, promoting tissue repair, and improving patient prognosis, which holds broad prospects.

## 5 Postoperative gastrointestinal hormone response and anti-inflammatory regulation

Postoperative reduction in appetite perception is closely related to various physiological and immune factors. In recent years, an increasing number of studies have revealed the important roles of gastrointestinal hormones such as Ghrelin (Gajewska et al., 2023), GLP-1 (Morrow et al., 2024), and PYY (Alosi and McFadden, 2009) in appetite perception regulation and inflammatory responses (Figure 1d) (Table 2). In the context of postoperative inflammatory stress, these changes in gastrointestinal hormones may represent an active adaptive mechanism of the body aimed at coping with postoperative inflammation and stress responses.

**Table 2:** Three postoperative gastrointestinal hormone responses and anti-inflammatory regulation.

Types	Model/Objective	Clinical outcomes	References
Ghrelin	Multiple immune cells and chronic inflammation models	Deletion of Ghrelin or GHSR → Macrophage polarization, and T cell activation ↓	Noh et al. (2022)
	C57BL / 6 J with HFD, myelin-specific Ghsr-KO (LysM-Cre)	System IL-6, TNF-α ↓, Macrophage NF-κB translocation ↓, Glycolysis ↓, Fatty acid oxidation ↑, Improvement of insulin resistance	Kim et al. (2024)
	*Ghsr* whole-body knockout & [D-Lys^3^]-GHRP-6 antagonist, Echinococcosis liver infection model	Number of infection foci ↓, IL-2, IFN-γ ↓, IL-4, IL-10 ↑, MyD88 / NF-κB / iNOS ↓	Zhu et al. (2025)
	RAW264.7 & BMDM: Ghsr-KO / siPrkaca (LPS)	Nuclear translocation of NF-κB ↓, TNF-α, IL-6↓, Oxidative metabolism ↑, and lipolysis ↓	Kim et al. (2024)
GLP-1	The nervous, cardiovascular, and musculoskeletal systems	GLP-1 / GLP-1 receptor agonist → Various inflammations ↓, Delays gastric emptying, suppresses appetite → Regulates energy balance	Zheng et al. (2024)
	Macrophages, T cells, etc.	GLP-1 receptor agonist inhibits TNF-α, IL-1β, and IL-6, and increases IL-10. Activate the STAT3 pathway and downregulate NF-κB.	Bendotti et al. (2022)
	Macrophages, Diabetic mice	GLP-1 receptor agonist → Macrophage polarization, and reduces IL-1β / IL-6 in myocardial and adipose tissues, tissue inflammation ↓	Alharbi (2024)
PYY	Roux-en-Y gastric bypass (RYGB)	YGB → PYY (1–36) ↑ → Improvement of pancreatic β-cell function, Local inflammation ↓	Guida and Ramracheya (2020)
	Subtotal gastrectomy for gastric cancer	Four months after the operation → PYY levels ↑, AUC ↑	Jung et al. (2022)
	1-cell differentiation, FFAR2^−/−^ mice	short-chain fatty acids (SCFA) → free fatty acid receptors 2 and 3 (FFAR2 / 3)-AMPK axis → PYY production ↑	Yu et al. (2024)
	Research models in mice and humans	SCFA → PYY & GLP-1 ↑, NF-κB-TNF-*α* / IL-6 ↓	Mazhar et al. (2023)
	Gut epithelial Paneth cells	PYY1-36 → Selectively destroys the mycelium of *C. albicans,* and mucosal inflammation ↓	Pierre et al. (2023)
	Nonimmune tissue, Monocyte system, Lymphocyte, Granulocyte	Y1R agonists → macrophage chemotaxis ↓, NF-κB translocation ↓	Chen et al. (2020)

Ghrelin, as an important gastrointestinal hormone, plays a key role in postoperative immune regulation and appetite perception modulation. Studies have found that Ghrelin promotes appetite perception through its receptor, growth hormone secretagogue receptor (GHSR) (Kim et al., 2024) and it also acts on immune cells, enhancing immune responses and promoting the function of immune cells, thereby linking metabolic status with immune responses (Mathur et al., 2020; Mingardi et al., 2024; Wu et al., 2024). Under normal circumstances, ghrelin secretion increases during fasting, stimulating appetite perception. However, postoperative stress and inflammatory responses significantly suppress ghrelin secretion. In the postoperative stress state, Ghrelin secretion is significantly reduced (by more than 50%), and this change alleviates inflammatory responses and maintains immune homeostasis through concerted mechanisms (Takata et al., 2015). Therefore, the decline in ghrelin levels in the postoperative stress state may represent an active adaptive response of the body to alleviate the burden on the immune system by reducing pro-inflammatory responses. In summary, Ghrelin as a metabolic-immune cross-regulatory factor, comprehensively regulates immune cell function by inhibiting pro-inflammatory cytokines, reprogramming immune cell metabolism, and multiple signaling pathways (Kim et al., 2024; Wu et al., 2024; Zhu et al., 2023). These mechanisms may help the body avoid excessive activation of the immune system, reducing the negative impacts of postoperative inflammation, thereby promoting recovery.

GLP-1, as an intestinal hormone, primarily regulates energy balance by suppressing appetite perception. Recent studies have shown that GLP-1 not only alleviates issues related to food intake by suppressing appetite perception after surgery but also exhibits significant anti-inflammatory effects (Zheng et al., 2024). GLP-1 modulates immune responses through various mechanisms, thereby reducing systemic inflammatory responses after surgery (Chang et al., 2024; VanderWielen and Brian Beam, 2024; Bendotti et al., 2022; Alharbi, 2024). Therefore, GLP-1 plays a role not only in appetite perception regulation but also in the modulation of postoperative immune responses, which should not be overlooked.

PYY, a multifunctional peptide hormone secreted by intestinal L cells, plays a central role in postoperative immune regulation and metabolic recovery. Apeptide-related hormones interact through complex interactions, forming a multi-layered immune regulatory network that not only collaborates in appetite perception regulation during postoperative recovery but also enhances the alleviation of postoperative inflammation through joint modulation of immune responses (Zhang et al., 2020; Zhu et al., 2024; Zhu et al., 2023). Clinical studies have shown that PYY levels significantly increase in postoperative patients, and through multiple pathways participate in postoperative inflammation alleviation and intestinal function repair (Guida and Ramracheya, 2020; Jung et al., 2022; Chen et al., 2020; Pierre et al., 2023). Appetite hormones can act on gut microbiota (Pierre et al., 2023), intestinal barrier function (Chen et al., 2023; Pierre et al., 2023), and the intestinal immune system (Christiansen et al., 2018; Mazhar et al., 2023), effectively weakening postoperative inflammatory responses and promoting recovery (Yu et al., 2024). Although existing studies have revealed the key roles of PYY in immune cell regulation and antibacterial defense, further exploration of its receptor subtype-specific interventions and clinical translation bottlenecks (such as short half-life) is still needed. Future development of Y1Y2 dual receptor agonists or nano-delivery systems may provide new strategies for treating postoperative complications.

## 6 Postoperative reduction in appetite perception and neuro-endocrine system regulation

The stress response triggered by surgical trauma activates the HPA axis, leading to the secretion of cortisol, which plays a central role in postoperative metabolic and immune homeostasis through dual regulatory mechanisms (Figure 1e). In terms of regulatory pathways, postoperative cortisol levels can rise to 3–5 times the baseline, and sustained high levels suppress appetite perception through both central and peripheral pathways (Durmisi et al., 2023; Okawa et al., 2024; Wagner et al., 2022). In terms of immune regulation, cortisol helps the body reduce the migration and activity of inflammatory cells during the postoperative recovery period, thereby alleviating both local and systemic inflammatory responses (Feng et al., 2024). On the other hand, it also prevents excessive activation of the immune system by inhibiting immune cell activation, which is crucial for preventing systemic inflammation triggered by postoperative stress (Sharma et al., 2024).

Furthermore, the hypothalamus-brainstem neuroendocrine network undergoes rewiring under inflammatory conditions, a process characterised by bidirectional reprogramming of NPY/AgRP inhibition and POMC/GLP-1 potentiation. Specifically, Pro-inflammatory cytokines, such as IL-1β and TNF-*α*, have been demonstrated to rapidly downregulate *Npy* transcription, thereby inhibiting approximately 35–40% of AgRP neurons (Reyes and Sawchenko, 2002; Chaves et al., 2020). This process effectively suppresses the hunger drive at its origin. Concurrently, pro-inflammatory cytokines activate POMC neurons via STAT3 and NF-κB signalling, augmenting α-MSH release and fortifying the MC4R-mediated satiation pathway (Jang et al., 2010; Chu et al., 2014). Concurrently, the ascending vagal-nucleus tractum loop has been shown to enhance the excitability of GLP-1 neurons in the brain stem. Blocking the central GLP-1 receptor (e.g., injection of Exendin-9-39) has been demonstrated to attenuate LPS-related anorexia significantly (Grill et al., 2004). This provides further evidence to support the hypothesis that GLP-1 is an inflammation-driven satiation amplifier. Conversely, a low energy intake has been demonstrated to inhibit the inflammatory immune response. Conversely, a low energy intake has been demonstrated to inhibit the inflammatory immune response.

The coordinated regulation of these neuroendocrine signals enables the body to rapidly lower the feeding threshold during acute inflammation, thus forming an adaptive neuroendocrine pattern with anti-hunger and satiety as the core features. Consequently, postoperative appetite perception loss may not be merely a passive response to surgical trauma, but rather a complex “active” adaptive neuroendocrine protective mechanism. This mechanism enables the body to achieve effective control of the inflammatory response after surgery by inducing a hypometabolic state, suppressing immune cell activity, and reducing sensitivity to proinflammatory signals.

## 7 Conclusions and perspectives

Postoperative reduction in appetite perception is a multifaceted protective response triggered by the body under physiological and psychological stress, integrating emotional arousal, affective processing, and cognitive appraisal of internal and external cues. This phenomenon is fundamentally reflective of an axis that encompasses emotion, perception, and affective cognition, orchestrated through the mechanisms of neuroendocrine signalling and metabolic reprogramming. The purpose of this orchestration is to achieve a fine-tuned balance among the processes of inflammation control, immune regulation, and tissue repair.

This process is not only marked by the activation of the HPA axis and cortisol release but also involves emotion-linked neuroendocrine modulation of peripheral metabolism, such as lipolysis, gastrointestinal hormone secretion (e.g., ghrelin, glucagon), and muscle catabolism. The subjective perception of appetite reduction, as filtered through emotional and cognitive networks, may represent an active self-protective strategy by the central nervous system to reduce tissue damage, suppress systemic inflammation, and support homeostatic recovery.

Traditional postoperative nutritional management has predominantly focused on the rapid restoration of food intake to accelerate recovery. However, this strategy often overlooks the neurocognitive and affective dimensions of appetite regulation. For patients experiencing strong inflammatory responses, excessive or premature supplementation may impose cognitive-metabolic mismatch, heighten inflammatory risk, and disrupt adaptive immune responses. Therefore, in clinical practice, appropriately moderated nutritional interventions respecting the patient’s appetite perception and emotional state may better support immune regulation and lower complication rates.

Future research should address three key areas: (i) Optimize postoperative nutritional management by developing interventions targeting the emotion-cognition-inflammation axis of appetite perception regulation. This includes pharmacological modulation of gut-brain-immune signaling and personalized feeding strategies. For elderly patients, protein supplementation programs should be tailored using sarcopenia risk assessment and integrated with resistance training to enhance metabolic and affective recovery. (ii) Establish dynamic, biomarker-based nutritional intervention models using stratification indicators such as IL-6 and GDF-15, aligned with patients’ cognitive-emotional status and appetite-related perception changes. (iii) Explore the interplay between neuroendocrine circuits, microbiota-derived signals, and affective-cognitive regulation to identify novel immune-metabolic control targets. Regarding the viewpoints we have proposed, we will conduct appetite and neuroimmune dynamic analyses on mice induced by trauma stimulation in a later stage to verify the perspectives we have put forward.

These approaches will help bridge the gap between biological perception and clinical action, ultimately facilitating a paradigm shift in postoperative care from empirical interventions to precision medicine guided by affective, perceptual, and cognitive parameters.
